# Supplementary material for: Surface-Initiated Polymerization with an Initiator Gradient: A Monte Carlo Simulation
Source: Polymers (Basel). 2024 Apr 25;16(9):1203. doi: 10.3390/polym16091203 (PMC11085584; doi:10.3390/polym16091203)
Supplement: Supplementary file 1 [file polymers-16-01203-s001.zip › polymers-2951198-supplementary.pdf]

Supporting Information

Surface-Initiated Polymerization with an Initiator Gradient: A Monte Carlo Simulation

Zhining Huang [1], Caixia Gu [1], Jiahao Li [1], Peng Xiang [1], Yanda Liao [2], Bang-Ping Jiang [1], Shichen Ji [1,3,*] and Xing-Can Shen [1,*]

[1] State Key Laboratory for Chemistry and Molecular Engineering of Medicinal Resources, Key Laboratory for Chemistry and Molecular Engineering of Medicinal Resources, Ministry of Education of China, Collaborative Innovation Center for Guangxi Ethnic Medicine, School of Chemistry and Pharmaceutical Sciences, Guangxi Normal University, Guilin 541004, China

[2] School of Computer Science and Engineering & School of Software, Guangxi Normal University, Guilin 541004, China

[3] State Key Laboratory of Molecular Engineering of Polymers, Fudan University, Shanghai 200433, China

* Correspondence: shichen.ji@mailbox.gxnu.edu.cn (S.J.); xcshen@mailbox.gxnu.edu.cn (X.-C.S.)

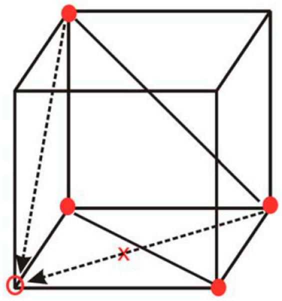

Figure S1. Illustration of two possible exchanges between a monomer and a vacancy, which involve bond intersections and are forbidden in this simulation.

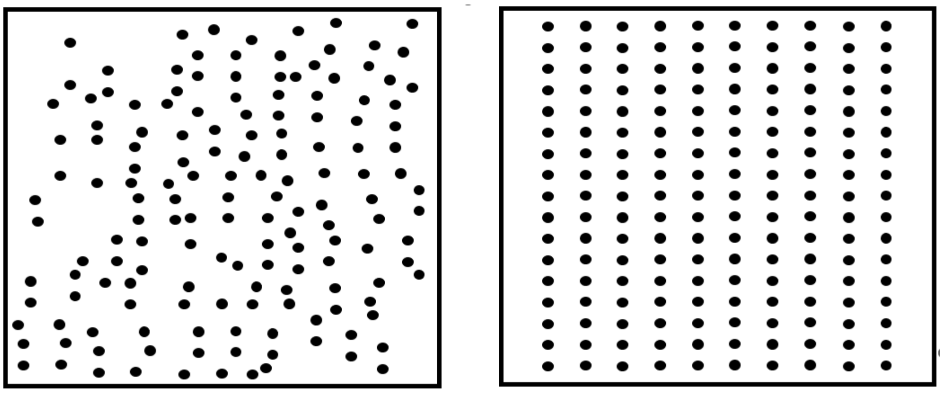

Figure S2. Illustration of initiators with a homogeneous distribution (left) and a regular distribution (right). Here, the homogeneous distribution means that all initiators are randomly placed on the substrate. While the regular distribution means that initiators are arranged in a certain lattice pattern.

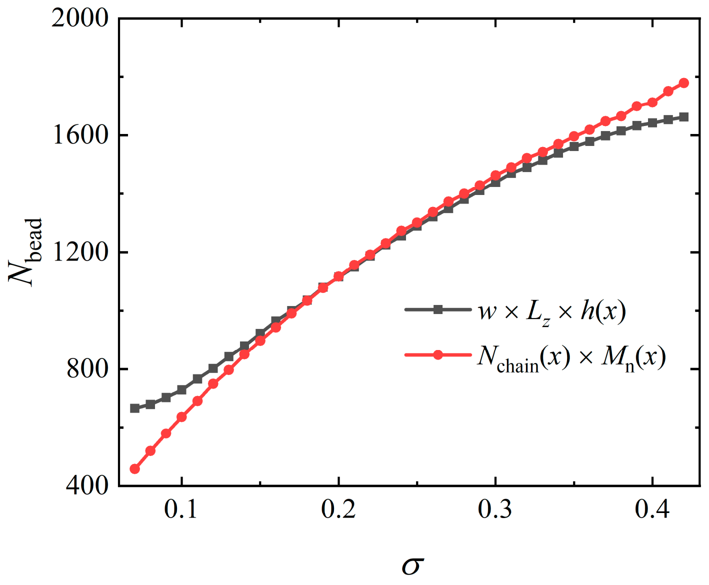

Figure S3. In surface-initiated polymerization with initiator gradient, at a given stripe, the number of free monomers consumed by corresponding initiators (red circles), and the number of actual beads of polymers above the stripe (black squares). The former equals the number of chains $N_{chain}(x)$ multiplied by the corresponding number-average molecular weight $M_n(x)$. While the latter equals the area ($w \times L_z$) multiplied by the corresponding dry height $h(x)$. A smaller number of polymer beads are found in the high grafting regions since the chains tend to the extend to lower grafting regions due to the unbalance lateral compression.
